# Stable Production of a Tethered Recombinant Eel Luteinizing Hormone Analog with High Potency in CHO DG44 Cells

**DOI:** 10.3390/cimb46060363

**Published:** 2024-06-15

**Authors:** Munkhzaya Byambaragchaa, Sei Hyen Park, Sang-Gwon Kim, Min Gyu Shin, Shin-Kwon Kim, Sung-Pyo Hur, Myung-Hum Park, Myung-Hwa Kang, Kwan-Sik Min

**Affiliations:** 1 Carbon-Neutral Resources Research Center, Hankyong National University, Anseong 17579, Republic of Korea; munkhzaya_b@yahoo.com (M.B.); pmh@tntresearch.co.kr (M.-H.P.); 2 Graduate School of Animal Biosciences, Hankyong National University, Anseong 17579, Republic of Korea; mrtree119@naver.com; 3 Aquaculture Research Division, National Institute of Fisheries Science, Busan 46083, Republic of Koreasmg159@korea.kr (M.G.S.); ksk4116@korea.kr (S.-K.K.); 4 Department of Marine Life Science, Jeju National University, Jeju 63243, Republic of Korea; hursp@jejunu.ac.kr; 5 Department of Food Science and Nutrition, Hoseo University, Asan 31499, Republic of Korea; mhkang@hoseo.edu; 6 Division of Animal BioScience, School of Animal Life Convergence Sciences, Institute of Genetic Engineering, Hankyong National University, Anseong 17579, Republic of Korea

**Keywords:** eel LH-M, Stable expression, CHO DG44 cells, cAMP response, pERK1/2

## Abstract

We produced a recombinant eel luteinizing hormone (rec-eel LH) analog with high potency in Chinese hamster ovary DG44 (CHO DG44) cells. The tethered eel LH mutant (LH-M), which had a linker comprising the equine chorionic gonadotropin (eLH/CG) β-subunit carboxyl-terminal peptide (CTP) region (amino acids 115 to 149), was inserted between the β-subunit and α-subunit of wild-type tethered eel LH (LH-wt). Monoclonal cells transfected with the tethered eel LH-wt and eel LH-M plasmids were isolated from five to nine clones of CHO DG44 cells, respectively. The secreted quantities abruptly increased on day 3, with peak levels of 5000–7500 ng/mL on day 9. The molecular weight of tethered rec-eel LH-wt was 32–36 kDa, while that of tethered rec-eel LH-M increased to approximately 38–44 kDa, indicating the detection of two bands. Treatment with the peptide N-glycanase F decreased the molecular weight by approximately 8 kDa. The oligosaccharides at the eCG β-subunit O-linked glycosylation sites were appropriately modified post-translation. The EC_50_ value and maximal responsiveness of eel LH-M increased by approximately 2.90- and 1.29-fold, respectively, indicating that the mutant exhibited more potent biological activity than eel LH-wt. Phosphorylated extracellular regulated kinase (pERK1/2) activation resulted in a sharp peak 5 min after agonist treatment, with a rapid decrease thereafter. These results indicate that the new tethered rec-eel LH analog had more potent activity in cAMP response than the tethered eel LH-wt in vitro. Taken together, this new eel LH analog can be produced in large quantities using a stable CHO DG44 cell system.

## 1. Introduction

Luteinizing hormone (LH), a member of the glycoprotein hormone family, is composed of a common α-subunit and unique β-subunits [1,2]. Chorionic gonadotropin (CG) β-subunit is specifically secreted from the placenta of primates and horses during early pregnancy and evolved from the LH β-subunit gene [3]. Human CG (hCG) β-subunit and hLH β-subunit genes are found to differ; however, a single gene encodes both the CG β-subunit and LH β-subunit in horses [4,5,6,7,8,9]. These glycoproteins are secreted from the pituitary gland and control gonadal function in mammals and fish species [10,11]. Eel LH α- and β-molecules are bound together via non-covalently linked subunits, which comprise 93 and 116 amino acids, respectively. The eel α-subunit has two N-linked glycosylation sites at positions Asn^56^ and Asn^79^; these glycosylation sites are highly conserved. The LH β-subunit has one glycosylation site at Asn^10^, as found in most mammals [12].

The oligosaccharide chains in LH, follicle-stimulating hormone (FSH), equine CG (eCG), and hCG are very important for cAMP/PKA signal transduction via receptors [13,14,15,16,17]. In fact, the glycosylation sites in eCG, eel FSH, and eel LH are suggested to be indispensable for biological activity through receptors in vitro and in vivo [11,18,19]. Rec-eel LH and FSH proteins are produced in the silkworm system; however, the *Bombyx mori* system does not have excellent biological activity for glycosylation, a post-translational modification, despite large quantities of rec-proteins [20,21]. Almost the same results are obtained in *Drosophila S2* cells [22] and *Pichia pastoris* [23,24]. These glycoproteins can be produced in Chinese hamster ovary (CHO)-K1 and CHO-suspension (CHO-S) cells; however, they cannot be produced on a large scale [2,18,19,25,26].

Owing to glycoprotein hormones, such as hCG, FSH, LH, and TSH, the CG β-subunit carboxy-terminal peptide (CTP) linker was found to induce substantially increased in vivo potency and circulatory half-lives [27,28,29,30,31,32]. Recently, eLH/CG β-subunit CTP linker attachments in tethered eel LH-wt and FSH-wt caused early secretion and signal transduction [25]. Remarkable results have been obtained in clinical trials with the hGH-MOD-4023 molecule, fused to hGH via the hCG β-subunit CTP linker (117–145 aa) [33,34,35,36].

Luteotropin/chorionic gonadotropin hormone receptor (LH/CGR) is a member of the superfamily of G protein-coupled receptors (GPCRs) and is involved in receptor-mediated responses, such as the cAMP response [37,38] and pERK1/2 activation [39,40,41]. Gαs proteins and β-arrestins are related to ERK signaling via two temporally distinct mechanisms: the G protein-dependent mechanism, which is rapid in onset, and the β-arrestin-dependent mechanism, which is slower in onset [42,43,44]. β-arrestins are involved in pERK1/2 with β-arrestin 1 and 2 in FSHR [45,46]. In terms of eel FSHR, pERK1/2 activation was found to be very fast, occurring 5 min after agonist stimulation [47,48]. Thus, eel LH should also be examined to elucidate its involvement in pERK1/2 activation and the cAMP signal response.

CHO DG44 cells are reported to be a good model for producing large quantities of rec-therapeutic proteins, such as erythropoietin [49], α-thrombin [50], alpha-1 antitrypsin [51], hFSH [52], hCG [53], and eLH/CG [54,55]. The Japanese eel is one of the most important fish species in East Asian countries. In general, the most common method for inducing maturation in female eels involves the weekly injection of freeze-dried pituitaries from Pacific salmon (SPE) and carp (CPE) [12,20,22]. Notably, SPE treatment does not completely induce oocyte maturation and ovulation and is considered a complete artificial aquaculture system for hormone composition. Thus, CHO DG44 cells were selected to produce large quantities of eel LH-M. As a result, attaching an eCTP linker between the β-subunit and α-subunit was found to induce more potent biological activity and a longer half-life.

Overall, a new potent hormone was found to be essential for artificial maturation, with rec-eel LH-M exhibiting more potent biological activity in vitro. Based on our results, a new rec-eel LH-M protein was continuously produced using a mass system in CHO DG44 cells.

## 2. Materials and Methods

### 2.1. Materials

The oligonucleotides were synthesized by Genotech (Daejeon, Republic of Korea). The pGEM-T easy cloning vector was purchased from Promega (Madison, WI, USA). The pOptiVEC^TM^-TOPO TA Cloning Kit, CHO DG44 cells, CD DG44 medium, CD FortiCHO^TM^ medium, CD OptiCHO^TM^ medium, Freedom^TM^ DG44 Kit, methotrexate (MTX) reagent, and cloning media were purchased from Life Technologies (Carlsbad, CA, USA). Freestyle MAX reagent and Lipofectamine-2000 were purchased from Invitrogen (Carlsbad, CA, USA). CHO-K1 cells and HEK 293 cells were obtained from the Korean Cell Line Bank (KCLB, Seoul, Republic of Korea). The mammalian expression vector, pCORON1000 SP VSV-G, was purchased from Amersham Biosciences (Piscataway, NJ, USA). CHO-S-SFM II medium, fetal bovine serum (FBS), Ham’s F-12 medium, and OptiMEM were purchased from Gibco (Grand Island, NY, USA). The monoclonal antibodies for western blotting and ELISA were produced in our laboratory [12] and labeled with horseradish peroxidase (HRP) by Medexx, Inc. (Seongnam, Republic of Korea). The PNGase F kit was purchased from New England Biolabs (Ipswich, MA, USA). The cAMP- homogeneous time-resolved fluorescence (HTRF) assay kit was purchased from Cisbio (Codolet, France). The Lumi-Light^plus^ western blotting substrate was obtained from Roche Inc. (Pleasanton, CA, USA). The pERK1/2 antibody, total ERK1/2 antibody, and goat anti-mouse HRP-conjugated secondary antibody were purchased from Cell Signaling Technology (Beverly, MA, USA). SuperSignal^TM^ West Pico PLUS Chemiluminescent substrate was purchased from Thermo Fisher Scientific Inc. (Waltham, MA, USA). DNA ligation reagents, endonucleases, polymerase chain reaction (PCR) reagents, and restriction enzymes were purchased from Takara Bio (Shiga, Japan). The QIAprep-Spin plasmid kit was purchased from Qiagen Inc. (Hilden, Germany), and disposable spinner flasks were purchased from Corning Inc. (Corning, NY, USA). Centriplus centrifugal filter devices were purchased from Amicon Bio Separations (Billerica, MA, USA). All other reagents were purchased from Sigma-Aldrich (St. Louis, MO, USA).

### 2.2. Vector Construction of Eel LH-wt and LH-M

**Figure 1:**
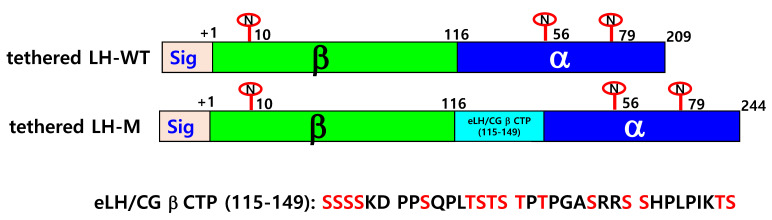
Schematic of wild-type tethered eel LH (LH-wt) and mutant tethered eel LH (LH-M) inserted eLH/CG β-subunit CTP region. The tethered form of eel LH β/α-wt was engineered to contain the β-subunit and common α-subunit sequences. The eLH/CG β-subunit carboxyl-terminal peptide linker was inserted between the β-subunit and α-subunit in the eel LH-M mutant using a polymerase chain reaction. The eLH/CG β-subunit CTP linker has 35 amino acids (115–149aa) and approximately 12 O-linked oligosaccharide sites. The numbers indicate the amino acids of the mature protein, except for the signal sequences. The encircled “N” denotes N-linked glycosylation sites at the eel LH β- and LH α-subunits, respectively. Green, FSH β; blue, α-subunit; and skyblue, eLH/CG β-subunit CTP linker. eLH/CG β CTP (115–149) represents the amino acid sequences of the eLH/CG β-subunit CTP linker. Red in the eLH/CG β CTP (115–149) denotes potential O-linked oligosaccharide sites.

We constructed a single-chain eel LH-wt a with a linked α-subunit without the signal sequence at the C-terminal region of the LH β-subunit, as described previously [11]. The LH-M mutant was also constructed by attaching an eCG β-subunit CTP region between the β-subunit and α-subunit via PCR, as described previously [25]. The full-length PCR products were ligated into the pGEMT-Easy vector. The full-length fragments of the tethered eel LH-wt and LH-M mutants were ligated into the pOptiVEC TOPO TA Cloning expression vector. Finally, the direction of insertion was confirmed via restriction enzyme cutting and sequencing for genetic verification. Figure 1 shows a schematic of tethered eel LH-wt and the LH-M mutant model, which has eLH/CG β-subunit CTP between the β-subunit and α-subunit.

### 2.3. Transfection into CHO DG44 Cells

Expression plasmids were linearized via Pvu 1 restriction enzyme cut and then transfected into CHO DG44 cells using FreeStyle^TM^ MAX reagent, according to the supplier’s protocol. Briefly, one day prior to transfection, CHO DG44 cells were passaged at a density of 3 × 10^5^ cells/mL. On the day of transfection, cell density was approximately 5 × 10^5^ cells/mL. For each transfection, 1.5 × 10^7^ viable CHO DG44 cells were transfected into a new 125-mL spinner flask. Pre-warmed, complete CD DG44 medium was added to the flask to a final volume of 30 mL. The plasmid DNA (18 µg) and 15 µL of FreeStyle^TM^ MAX Reagent were mixed with OptiPRO^TM^ SFM. The diluted samples were then incubated for 10 min at room temperature to induce complex formation. The DNA-FreeStyle^TM^ MAX reagent complex was added dropwise to the cells, with slow swirling of the flask.

### 2.4. Single Cell Isolation and Production of the Rec-Eel LH-wt and LH-M Proteins

At 48 h after transfection, the cells were transferred into complete CD OptiCHO^TM^ medium supplemented with 8 mM of l-glutamine. Fresh growth medium was replaced every 3–4 days for approximately 10–14 days. The cells were adapted for amplification by increasing the amount of MTX reagent. MTX-amplified cells were grown in a fresh growth medium without 8 mM l-glutamine. On the day of cloning, the diluted cells were dispensed at 0.5–2 cells per well into a 96-well plate. After isolation, single-cell colonies were transferred to 24-well plates, 6-well plates, and T-25 flasks. Finally, the clones were expanded in 125-mL shaker flasks at 37 °C and 8% CO_2_, with shaking at 130–135 rpm. To confirm protein production, single-clone cells were cultured in 30 mL of fresh medium supplemented with 4 mM l-glutamine. The supernatant was collected and analyzed to determine the presence of the tethered rec-LH-wt and LH-M proteins. Finally, the culture media were collected on day 9 post-seeding and centrifuged at 100,000× *g* for 10 min at 4 °C to remove cell debris. The supernatant was collected and frozen at −80 °C.

### 2.5. Quantitation and Western Blotting of the Rec-Eel LH Proteins

The collected proteins were quantified via double-sandwich enzyme-linked immunosorbent assay (ELISA) in plates coated with a monoclonal antibody, as described previously [11]. One hundred microliters of the medium were added to the plates, which were then incubated for 1–2 h at room temperature. Thereafter, HRP-conjugated anti-eel antibody was added for 1 h. The wells were washed 5 times, and 100 μL of substrate solution (tetramethylbenzidine) was added to the wells for 20 min. The absorbance of each well was measured at 450 nm using a microplate reader (Cytation 3; BioTek, Winooski, VT, USA).

For western blot analysis, the collected sample (20 µL) was subjected to 12% sodium dodecyl sulfate polyacrylamide gel electrophoresis (SDS-PAGE). The proteins were transferred onto a membrane, blocked with 5% skim milk, and then incubated with monoclonal antibodies overnight. After washing with TBS-T, the membrane was incubated with a HRP-conjugated anti-mouse secondary antibody for 2 h, followed by 2 mL of Lumi-Light substrate solution for 5 min. Detection was performed using an enhanced chemiluminescence system.

To enzymatically release *N*-linked oligosaccharides, modified glycans were removed from rec-proteins via treatment with the *N*-glycosylation enzyme. Briefly, the rec-protein (15 µg) was incubated for 1 h at 3 °C with PNGase F [1 µL enzymes (2.5 U/mL)/20 µL sample + 2 µL of 10 × Glycobuffer + 2 µL of 10% NP-40] after boiling at 100 °C for 10 min with 1 µL of 10 × Glycoprotein Denaturing Buffer. The samples were analyzed using SDS-PAGE, followed by western blot analysis.

### 2.6. Analysis of cAMP Levels via Homogenous Time-Resolved Fluorescence Assays

cAMP accumulation in CHO-K1 cells expressing eel LH/CGR was measured using a cAMP Dynamics 2 competitive immunoassay kit. Briefly, rec-LH ligand (5 μL) was added to each well and incubated for 30 min. The cryptate-conjugated anti-cAMP monoclonal antibody and d2-labeled cAMP reagent were added to each well, and then incubated for 1 h at room temperature. The compatible HTRF energy transfer (665 nm/620 nm) was measured using a TriStar2 S LB942 mi microplate reader (BERTHOLD Tech, Wildbad, Germany). The cAMP concentration for Delta F% values was calculated using the GraphPad Prism software (version 6.0; GraphPad Software Inc., La Jolla, CA, USA).

### 2.7. Phospho-ERK1/2 Time Course

The pCORON1000 SP VSV-G plasmid containing eel LH/CGR was transfected into HEK293 cells. After 48 h, the cells were starved for at least 4–6 h and then treated with an agonist. Cells were lysed using RIPA buffer (Sigma-Aldrich, St. Louis, MO, USA). Equal amounts of cellular extracts were loaded onto 10% SDS-PAGE gels and transferred onto nitrocellulose membranes. pERK1/2 and total ERK1/2 were detected via immunoblotting using rabbit polyclonal anti-phospho-p44/42 MAPK (1:2000) and anti-MAPK1/2 (1:3000), respectively. The membranes were then incubated with an anti-rabbit secondary antibody. Chemiluminescence was detected using SuperSignal^TM^ West Pico reagent, and phosphorylated ERK1/2 immunoblots were quantified via densitometry using Image-Lab (Bio-Rad, Hercules, CA, USA).

### 2.8. Data Analysis

Dose-response curves were generated for experiments performed in duplicate. GraphPad Prism 6.0 (San Diego, CA, USA) was used to analyze the cAMP response, EC_50_ values, and stimulation curves. Curves fitted in a single experiment were normalized to background signals measured in mock-transfected cells. The pERK1/2 values were calculated using GraFit Version 5 (Erithacus Software, Horley, Surrey, UK). The results are expressed as mean ± standard error of the mean of three independent experiments.

## 3. Results

### 3.1. Isolation of Single Cells after Transfection

Transfected cells were selected at the first stage using CD-OptiCHO medium for approximately 2–3 weeks. The viability of cells transfected with tethered eel LH-wt and eel LH-M plasmids decreased gradually to 36% and then continuously increased to 58%, 78%, and >90%. The selected cells were subjected to the second round of selection using the MTX reagent. Cell viability decreased to 40–50% due to the first treatment with 500 nM MTX. The lowest viability (16–31%) was obtained in 2 weeks via treatment with 500 nM MTX. Thereafter, a modest increase was recorded for another two weeks. The MTX concentration was increased by 2 µM to boost the gene copy number. However, cell viability did not decrease below 80%, indicating a slight decrease with recovery to over 90% in two weeks. The MTX-selected cells were then isolated using a complete cloning medium in 96-well plates. Finally, monoclonal cells expressing tethered rec-eel LH-wt and eel LH-M were isolated from five and nine clones, respectively.

### 3.2. Secreted Quantity of the Tethered Rec-Eel LH-wt Protein

**Figure 2:**
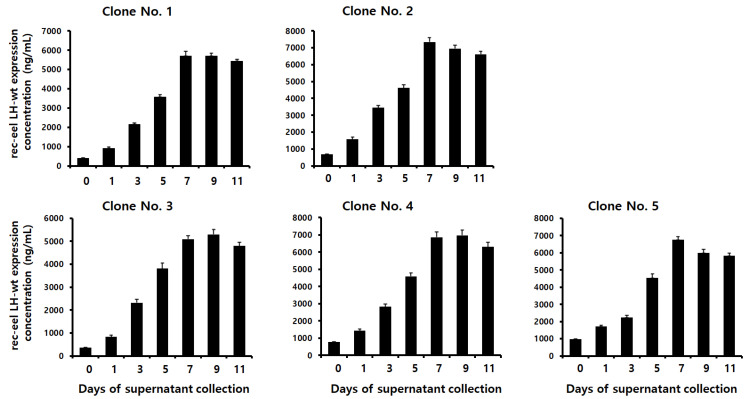
Quantity of rec-eel LH-wt secreted from CHO DG44 cells based on the day of culture. Supernatant was collected on days 0, 1, 3, 5, 9, and 11 of culture. The expression levels of the tethered rec-eel LH-wt protein were analyzed using a sandwich enzyme-linked immunosorbent assay, as described in the Methods section. Data for five clones are presented as representatives. Values are expressed as mean ± standard error of mean of at least three independent experiments.

The quantity of the tethered rec-eel LH-wt protein secreted from five clones was determined using ELISA. Supernatants were collected on days 1, 3, 5, 7, 9, and 11 post-cultivations. Tethered rec-eel LH-wt was low on day 1 post-culture and started to increase on day 3. The concentrations gradually increased until the final supernatant was collected, as shown in Figure 2. The amount of tethered rec-LH-wt ranged from 2150 to 3450 ng/mL on day 3. The expression levels increased to 3578–4625 ng/mL on day 5. High amounts of 7346 ± 246 and 6868 ± 298 ng/mL were detected in clones 2 and 4 on day 7, respectively. The secreted quantities of both clones were highest on days 7 and 9; however, high concentrations were consistently observed until day 11 for all clones. These results indicated that the expression level of tethered eel LH-wt gradually increased with cultivation time, with optimal recovery on day 9 in CHO-DG44 cells.

### 3.3. Western Blotting for Tethered Rec-Eel LH-wt

**Figure 3:**
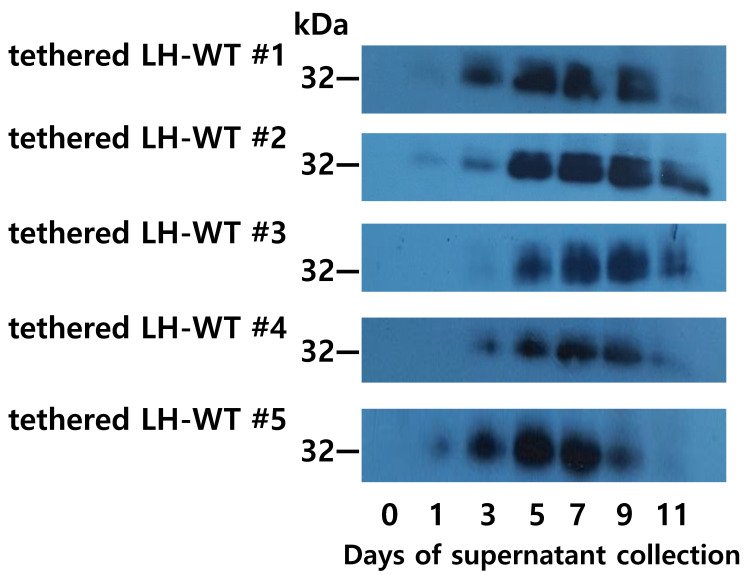
Western blot analysis of the tethered rec-eel LH-wt proteins produced from single cells. Supernatants from the 5 colonies were collected on the day of cultivation. Five colonies (tethered eel LH-wt 1 to LH-wt 5) were selected, and 20 μL of supernatant was used for sodium dodecyl sulfate-polyacrylamide gel electrophoresis. The proteins were then blotted onto a membrane and detected using a monoclonal antibody (anti-eel α-subunit) and HRP-conjugated goat anti-mouse IgG antibodies.

The molecular weight of tethered rec-eel LH-wt was determined via western blot analysis using the anti-eel glycoprotein hormone α-subunit monoclonal antibody, as described previously [12]. The collected supernatants (20 µL) were analyzed using western blotting. Specific bands were detected at 32–36 kDa in all samples (Figure 3), and weak bands were detected in all clones on day 3. The band intensity steadily thickened, and the strongest signal was observed on days 7–9, with a slight decrease on day 11. According to the ELISA and western blot results, optimal recovery occurred on days 7 and 9.

### 3.4. Secreted Quantity of the Tethered Rec-Eel LH-M Protein

**Figure 4:**
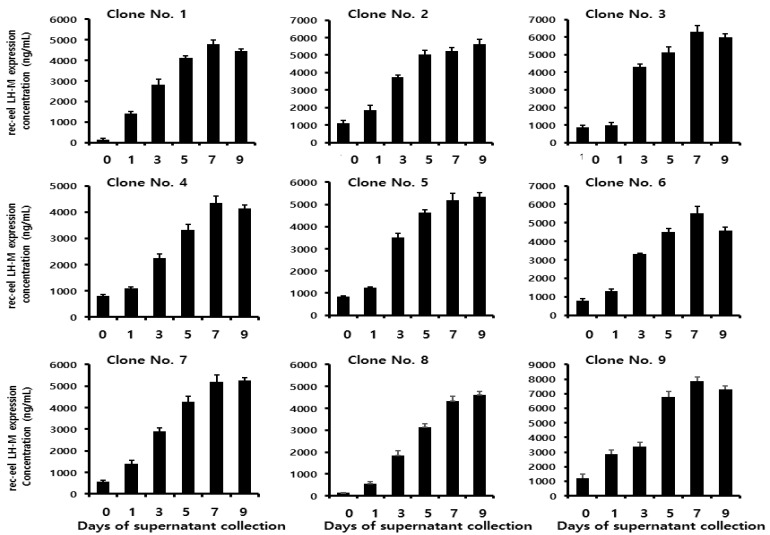
Quantity of the tethered rec-eel LH-M proteins secreted from CHO-DG44 cells on the day of culture. Supernatants were collected on days 0, 1, 3, 5, 7, and 9 post-cultivations. Nine clones were isolated, and their expression levels were determined in a time-dependent manner. The expression level was analyzed using a sandwich enzyme-linked immunosorbent assay, as described in the Methods section. Values are expressed as mean ± standard error of mean of at least three independent experiments.

To analyze the quantity of secreted proteins in the nine clones of the tethered eel LH-M mutant, the supernatant was collected on days 1, 3, 5, 7, and 9 post-cultivations. The quantity of the tethered rec-eel LH-M protein gradually increased, as shown in Figure 4. Based on the clones, 543–2847 ng/mL of the secreted protein was detected on day 1. The secreted pattern was almost the same as that detected in eel LH-wt. The secreted levels rapidly increased to 3332–6763 ng/mL on day 5 and reached 4341–7865 ng/mL on days 7 and 9. The expression was maintained until day 9. The highest expression level of 7865 ± 278 ng/mL was obtained on day 7 in clone 9. The secreted quantities were high on days 7 and 9 post-cultivation in all clones.

### 3.5. Western Blotting for Tethered Rec-Eel FSH-M

**Figure 5:**
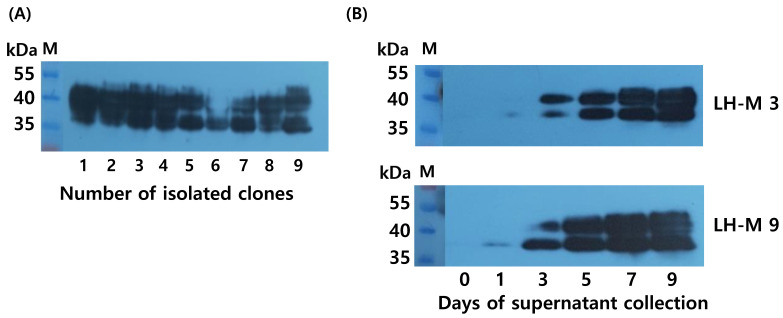
Western blot analysis of the tethered rec-eel LH-M proteins produced from monoclonal cells. Supernatants from nine colonies were collected on the day of cultivation. The samples were prepared for sodium dodecyl sulfate-polyacrylamide gel electrophoresis, and membranes were detected using specific monoclonal antibodies (anti-eel α-subunit). In (**A**), 20 μL of sample collected on day 9 was loaded into the wells. Two specific bands were detected in all samples. (**B**) Two colonies (eel LH-M 3 and LH-M 9) were selected for western blot analysis, and 20 μL of the supernatant collected on the day of culture was used for western blot analysis.

The tethered eel LH-M protein was isolated from nine clones. Briefly, 20 µL of supernatant was collected on day 9 for western blotting. Two specific bands were detected at 38–44 kDa, as shown in Figure 5A. The molecular weight of tethered rec-eel LH-M increased by approximately 6–8 kDa compared to that of tethered eel LH-wt. The addition of the eLH/CG CTP β-subunit linker plays a pivotal role in glycosylation. Thus, the secretion pattern was detected according to the culture time of clones 3 and 9, which indicated gradual increases in the band intensity (Figure 5B). Weak bands were detected on day 3, and strong signals were detected on days 7 and 9, indicating the strong detection of two specific bands. Two bands with different molecular weights are presumed to be due to the modification of the oligosaccharide chains.

### 3.6. Deglycosylation of Rec-Eel LH-wt and LH-M Proteins

**Figure 6:**
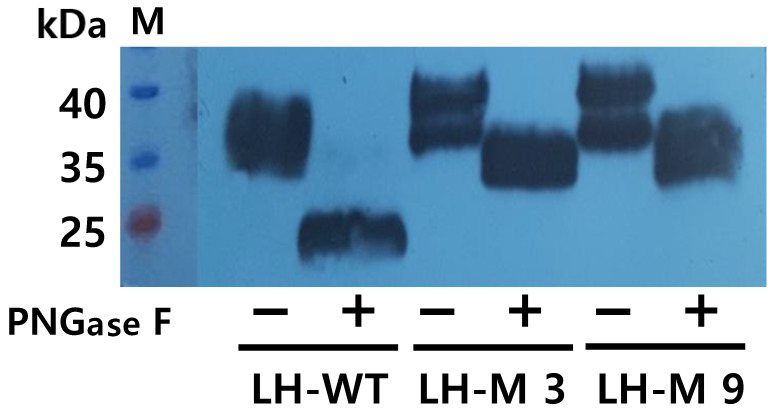
Deglycosylation of the tethered eel LH-wt and LH-M proteins. The proteins from tethered eel LH-wt, LH-M 3, and LH-M 9 were treated with peptide-N-glycanase F to remove N-linked oligosaccharides, and western blot analysis was performed. The molecular weights of the tethered rec-eel LH-wt and LH-M proteins decreased by approximately 8–10 kDa.

The molecular weights of tethered rec-eel LH-wt and the LH-M mutant were approximately 32–35 and 38–44 kDa, respectively, as shown in Figure 6. However, treatment of the tethered rec-eel LH-wt with PNGase F reduced its molecular weight to approximately 24–28 kDa, indicating a decrease of approximately 8–9 kDa. To further characterize tethered rec-eel LH-M, treatment with the PNGase F enzyme markedly decreased the molecular weight to 32–36 kDa. The eLH/CG CTP β-subunit linker region has only 35 amino acids, including 12 potential O-linked glycosylation sites. Therefore, the 6–8 kDa increase in molecular weight indicates a partial modification of additional oligosaccharides in the O-linked glycosylation sites. Thus, the carbohydrate content of tethered rec-eel LH-wt and eel LH-M proteins from CHO DG44 cells was appropriately modified.

### 3.7. Biological Activities of the Tethered Rec-Eel LH-wt and LH-M Proteins

**Figure 7:**
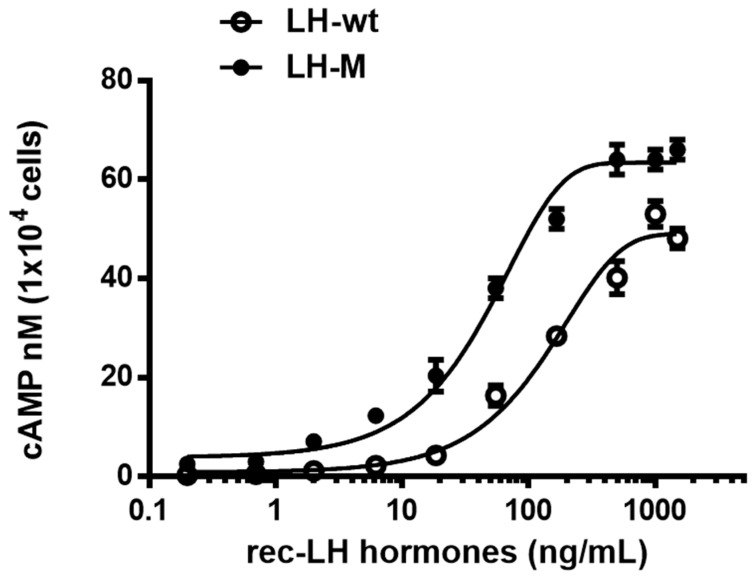
Effect of the tethered rec-eel LH-wt and LH-M proteins on cyclic adenine monophosphate (cAMP) production in cells expressing the eel luteinizing hormone receptor. CHO-K1 cells transiently transfected with eel LHR were seeded in 384-well plates (10,000 cells/well) at 24 h post-transfection. The cells were activated with the tethered rec-eel LH-wt and LH-M proteins by increasing concentrations (0–1500 ng/mL) by ELISA analysis for 30 min at room temperature. cAMP production was detected using a homogeneous time-resolved fluorescence assay and presented as delta F%. cAMP concentrations were calculated using GraphPad Prism software. Each data point represents mean ± standard error of mean of triplicate experiments. The mean data were fitted to an equation to derive a single-phase exponential decay curve.

The in vitro biological activity was assessed using transfected CHO-K1 cells expressing the LH/CG receptor. The potency of cAMP activation in tethered rec-eel LH-wt and LH-M mutants is shown in Figure 7. The dose-response curve of the tethered eel LH-M mutant slightly shifted to the left compared to that of tethered eel LH-wt. Tethered rec-eel LH-wt exhibited full biological activity, with an EC_50_ value of 138.8 ng/mL and Rmax of 49.0 ± 1.2 nM/10^4^ cells. However, the biological activity of tethered eel LH-M was higher than that of tethered LH-wt, with an EC_50_ and Rmax values of 47.8 ng/mL and 63.4 ± 1.5 nM/10^4^ cells (Table 1), indicating a 2.9- and 1.29-fold increase, respectively. This increase enables the attachment of the eLH/CG β-subunit CTP linker, which contains approximately 12 *O*-linked glycosylation sites.

### 3.8. Phospho-ERK1/2 Activation

**Figure 8:**
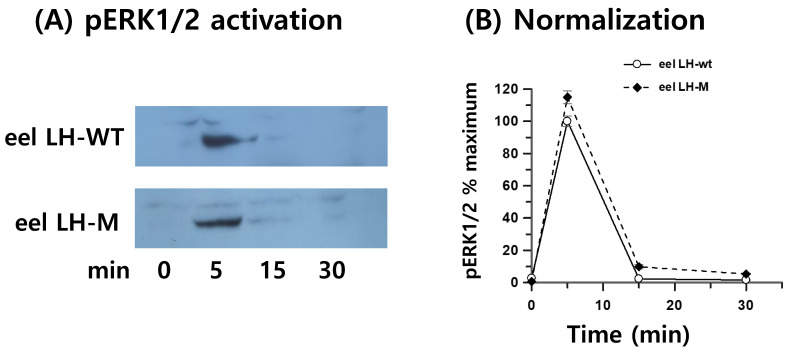
pERK1/2 activation stimulated by the eel FSH receptor. (**A**) Western blot results for pERK1/2. (**B**) Western blot results normalized as maximal response % (100% for eel LH-wt at 5 min). The eel LH receptor cDNA was transiently transfected into HEK293 cells, which were starved for 4–6 h and then stimulated with 400 ng/mL agonists for the indicated times. Whole cell lysates were analyzed to determine the levels of pERK1/2 and total ERK. Twenty micrograms of protein were loaded into each sample lane. The pERK1/2 band was quantified via densitometry, and pERK1/2 was normalized to the level of total ERK. Representative data are shown, and the graphs contain the mean ± SE values of three independent experiments. No significant differences were observed between the curves of the tethered eel LH-wt- and LH-M-treated samples.

MAPK activation sharply increased (within 5 min) in HEK 293 cells transiently transfected to express eel LH/CGR and stimulated with tethered rec-eel LH and rec-eel LH-M (Figure 8). Rapid activation of pERK1/2 was observed at 5 min, which then decreased abruptly. After agonist stimulation, pERK1/2 activity was highest at 5 min and decreased to approximately 10% of the maximal level at 15 min and 30 min. No difference was observed between the tethered eel LH-wt and LH-M groups. These results indicate that eel LH/CGR by a rec-LH agonist induces PKA-dependent pERK1/2 activation. However, the roles of PKA and PKC in pERK1/2 need to be elucidated.

## 4. Discussion

Based on the results of the present study, the attachment of an eLH/CG β-subunit-CTP linker between the β-subunit and α-subunit of tethered eel LH significantly increases the biological activity of cAMP responsiveness in vitro. Moreover, this new eel LH analog could be produced in large quantities using a stable CHO DG44 cell system. In cells expressing the eel LH receptor, tethered eel LH-M displayed more potent activity in the PKA signal transduction pathway than tethered eel LH-wt. The eLH/CG β-subunit CTP regions are essential in glycoprotein hormones to achieve early expression, a novel long-acting rec-protein hormone analog, and more potent biological activity.

In the present study, the highest secreted quantity of 5000–7500 ng/mL was obtained on day nine post-cultivation. The expression level increased by approximately 10–14-fold compared to the transient expression in CHO-K1 and CHO-S cells, as described previously [11,25]. However, these levels are markedly lower than those obtained in previous studies on human α-thrombin (1.5 g/L) and human alpha-1 antitrypsin (1.05 g/L) by methotrexate (MTX) amplification [49,50,51]. Therefore, the rec-protein quantity in CHO DG44 cells may depend on the gene of interest. A strong band was also detected via western blotting, despite loading only 20 µL of the supernatant.

Based on western blot analysis, the molecular weights of tethered rec-eel LH-wt and LH-M were 32–36 kDa and 38–44 kDa, respectively. These results are consistent with those of a recent study, in which the same broadband was observed for tethered eel LH-M transiently produced from CHO-S cells, indicating the detection of two specific bands [25]. Therefore, the dimeric eel LH α/β linked with a non-covalent bond from the eel pituitary was approximately 32–38 kDa [56]. Based on these results, broad bands are widely detected for tethered eel LH-M; therefore, the increased molecular weight was due to the attachment of the eCG β-subunit CTP linker. The molecular weight following PNGase F treatment clearly decreased by approximately 8 kDa. These results are almost consistent with those of previous studies, suggesting that the purified eel α-subunit from eel pituitary reacted with the 17 and 19 kDa proteins; however, the molecular weight decreased to 13 kDa owing to N-glycopeptidase F treatment [22].

In previous studies on hCTP and eCTP linker attachment in glycoprotein hormones, the molecular weight and biological activity of hFSH β-hCTP-α, hCTP-hGH-hCTP-hCTP, hTSH β-hCTP-α, [27,33,57], and eel FSH-M [25] were found to increase. These results are consistent with our findings, as the molecular weight of eel LH-M increased by approximately 6–8 kDa. Therefore, the linker containing O-linked glycosylation sites increases the molecular weight, indicating higher PKA/cAMP responsiveness of rec-eel LH-M than eel LH-wt. However, the EC_50_ value and maximal cAMP responsiveness stimulation markedly decreased in the deglycosylated eel LH mutants in a dose-dependent manner in vitro [11]. Thus, the oligosaccharide chains of the glycoprotein hormones may contribute to their biological activity and molecular weight. hCTP-hGH-hCTP-hCTP was developed as a long-acting human GH for once-weekly administration to GH-deficient adults and children [33,36,58]. Although we did not present in vivo results in this experiment, the addition of the eLH/CG β-subunit CTP linker will probably enhance the hormone’s half-life through post-translational modification of the CTP region. In general, eel males need to inject hormones (SPE and hCG) once a week for a long time (7–8 weeks) to induce maturity. Therefore, in vivo experiments are the most important to utilize these recombinants for the induction of sexual maturation in eel males. Nevertheless, we suggest that the eCTP linker attachment contributes to the biological activity and long-acting functions of proteins in vivo because of the increased molecular weight.

The eLH/CG β-subunit CTP linker region contains 34 amino acids, several threonine/serine residues for potential post-phosphorylation, and approximately 12 O-linked glycosylation sites. The O-linked glycosylation induces a specific conformation. According to previous studies, the O-linked glycosylation sites in eCG and hCG play indispensable roles in the secretion of wild-type hormones into the cell supernatant, biological activity, and half-life [1,2,16,25,59]. However, these investigators also insisted that the hCG CTP regions are not important for receptor binding and signal transduction in vitro. Nevertheless, the CTP regions are dispensable for biological activity and half-life in vivo [59]. Deletion of the eLH/CG β-subunit from the 34 amino acids, including the O-linked glycosylation sites, significantly delayed the secretion time in CHO-S cells [19]. Thus, attachment of the eLH/CG β-subunit CTP linker to the eel LH-M molecule may prolong the circulating half-life of the rec-protein. Therefore, the eel LH-M mutant with the eLH/CG β-subunit CTP linker plays a pivotal role in signal transduction through the receptors.

Phosphorylation of ERK1/2 proceeds via the sequential activation of three kinases, Raf1, MEK1, and ERK1/2 [60,61]. The intracellular signaling pathway is activated by LH and includes the Gs-mediated activation of adenylate cyclase, resulting in cAMP production and PKA activation in a cell-specific and G protein-dependent manner [62,63]. This ERK1/2-mediated regulatory process has been observed for many GPCRs that interact with β-arrestins, such as V2 vasopressin receptor [64], parathyroid hormone receptor [65], β2 adrenergic receptor [43], u-opioid receptor [66], neurotensin receptor 1 [44], glucagon-like peptide-1 receptor [67], and angiotensin II [68]. In the present study, pERK1/2 exhibited a peak response 5 min after agonist stimulation. These results are consistent with FSH- and hCG-stimulated pERK1/2 activation in a dose-dependent manner, with a peak observed at approximately 6 min [46,62]. Based on our results, pERK1/2 is activated in almost the same manner as most GPCRs. Taken together, stimulation of the eel LH/CGR-mediated signaling pathway leads to pERK1/2 activation. pERK1/2 activation by LH/CGR-mediated signaling must be further studied to determine whether a new pathway involving the phosphorylation of downstream effectors of the MAPK pathway exists.

## 5. Conclusions

Herein, tethered rec-eel LH-M was found to exhibit more potent biological activity for cAMP responsiveness than tethered rec-eel LH-wt in cells expressing eel the LH/CG receptor. Single cells expressing the tethered rec-eel LH-wt and LH-M proteins were isolated from CHO DG44 cells. These stable cells secreted approximately 15-fold more protein than CHO-S cells. The molecular weight of tethered rec-eel LH-M ranged from 38 to 44 kDa, and two specific bands were detected. The cAMP responsiveness (EC_50_ value) in tethered eel LH-M was increased by 2.90-fold, and the maximal responsiveness was 1.29-fold higher than that of tethered eel LH-wt. Similar activation of pERK1/2 levels was recorded with a sharp peak at 5 min, followed by a rapid decline. These findings are markedly important to the production of new potent analogs in large quantities using a stable CHO DG44 cell system. The eLH/CG β-subunit CTP linker containing 12 O-linked glycosylation sites needs to exhibit potent activity in vitro and is required for maximal cAMP responsiveness to enable efficient activation of the pERK1/2 signal transduction pathway. In addition, an in vivo study on the effect of inducing sexual maturity must be presented for the tethered rec-eel LH-M. Currently, in vivo research to induce male maturity is being conducted in collaboration with a specialized research institute.

## Figures and Tables

**Table 1:** Bioactivity of rec-eel LH proteins in cells expressing eel LH receptor.

Rec-LH Hormones	cAMP Responses		
	Basal *^a^* (nM/10^4^ Cells)	Log (EC_50_) (ng/mL)	Rmax *^b^* (nM/10^4^ Cells)
LH-wt	0.9 ± 0.5 *	138.8 * (116.6 to 171.4) *^c^*	49.0 ± 1.2 *
LH-M	2.1 ± 0.8 *	47.8 ** (40.1 to 57.4)	63.4 ± 1.5 **

Values are the means ± SEM of triplicate experiments. Log (EC_50_) values were determined from the concentration-response curves from in vitro bioassays. *^a^* Basal cAMP level average without agonist treatment. *^b^* Rmax average cAMP level/10^4^ cells. *^c^* Geometric mean (95% confidence intervals) of at least three experiments. *, ** Values with different superscripts are significantly different (*p* < 0.05).

## Data Availability

Data is contained within the article.
